# Surveillance and Genetic Characterization of Virulent Newcastle Disease Virus Subgenotype V.3 in Indigenous Chickens from Backyard Poultry Farms and Live Bird Markets in Kenya

**DOI:** 10.3390/v13010103

**Published:** 2021-01-13

**Authors:** Henry M. Kariithi, Helena L. Ferreira, Catharine N. Welch, Leonard O. Ateya, Auleria A. Apopo, Richard Zoller, Jeremy D. Volkening, Dawn Williams-Coplin, Darren J. Parris, Tim L. Olivier, Dana Goldenberg, Yatinder S. Binepal, Sonia M. Hernandez, Claudio L. Afonso, David L. Suarez

**Affiliations:** 1 Exotic and Emerging Avian Viral Diseases Research Unit, Southeast Poultry Research Laboratory, U.S. National Poultry Research Center, USDA-ARS, 934 College Station Road, Athens, GA 30605, USA; henry.kariithi@usda.gov (H.M.K.); hlage@usp.br (H.L.F.); ricky.zoller@usda.gov (R.Z.); dawn.williamscoplin@usda.gov (D.W.-C.); darren.parris@usda.gov (D.J.P.); tim.olivier@usda.gov (T.L.O.); Danagold75@gmail.com (D.G.); 2 Biotechnology Research Institute, Kenya Agricultural and Livestock Research Organization, Kaptagat Road, Loresho, Nairobi P.O. Box 57811-00200, Kenya; leonard.ateya@kalro.org (L.O.A.); yatinder.binepal@kalro.org (Y.S.B.); 3 Department of Veterinary Medicine, FZEA-USP, University of Sao Paulo, Pirassununga 13635900, Brazil; 4 Florida Department of Environmental Protection, Division of Recreation and Parks 33104 NW 192nd Ave, Okeechobee, FL 34972, USA; catharine.welch@dep.state.fl.us; 5 Directorate of Veterinary Services, State Department for Livestock, Ministry of Agriculture, Livestock, Fisheries and Cooperatives, Nairobi P.O. Box 34188-00100, Kenya; aajiambo@gmail.com; 6 BASE2BIO, Oshkosh, WI 54904, USA; jeremy.volkening@base2bio.com; 7 Warnell School of Forestry and Natural Resources and The Southeastern Cooperative Wildlife Disease Study at the College of Veterinary Medicine, University of Georgia, Athens, GA 30602, USA; shernz@uga.edu

**Keywords:** oropharyngeal, cloacal, Newcastle disease, spatial-temporal dispersal, phylogeography

## Abstract

Kenyan poultry consists of ~80% free-range indigenous chickens kept in small flocks (~30 birds) on backyard poultry farms (BPFs) and they are traded via live bird markets (LBMs). Newcastle disease virus (NDV) was detected in samples collected from chickens, wild farm birds, and other domestic poultry species during a 2017–2018 survey conducted at 66 BPFs and 21 LBMs in nine Kenyan counties. NDV nucleic acids were detected by rRT-PCR L-test in 39.5% (641/1621) of 1621 analyzed samples, of which 9.67% (62/641) were NDV-positive by both the L-test and a fusion-test designed to identify the virulent virus, with a majority being at LBMs (64.5%; 40/62) compared to BPFs (25.5%; 22/62). Virus isolation and next-generation sequencing (NGS) on a subset of samples resulted in 32 complete NDV genome sequences with 95.8–100% nucleotide identities amongst themselves and 95.7-98.2% identity with other east African isolates from 2010-2016. These isolates were classified as a new sub-genotype, V.3, and shared 86.5–88.9% and 88.5–91.8% nucleotide identities with subgenotypes V.1 and V.2 viruses, respectively. The putative fusion protein cleavage site (^113^R-Q-K-R↓F ^117^) in all 32 isolates, and a 1.86 ICPI score of an isolate from a BPF chicken that had clinical signs consistent with Newcastle disease, confirmed the high virulence of the NDVs. Compared to genotypes V and VI viruses, the attachment (HN) protein of 18 of the 32 vNDVs had amino acid substitutions in the antigenic sites. A time-scaled phylogeographic analysis suggests a west-to-east dispersal of the NDVs via the live chicken trade, but the virus origins remain unconfirmed due to scarcity of continuous and systematic surveillance data. This study reveals the widespread prevalence of vNDVs in Kenyan backyard poultry, the central role of LBMs in the dispersal and possibly generation of new virus variants, and the need for robust molecular epidemiological surveillance in poultry and non-poultry avian species.

## 1. Introduction

Village chicken production is important in household and national economies of most African countries [1]. Indigenous chickens (*Gallus gallus domesticus*), which, in the Kenyan context, are described as “non-descript crosses of Asiatic meat and game types, Mediterranean egg-types and Bantams of various origins” [2], dominate poultry production systems (backyard poultry farms; BPFs) in rural Kenya, accounting for ~ 80% of the poultry population and approximately 50% of egg production. Live poultry trade between the BPFs and live bird markets (LBMs) is highly unregulated, and biosecurity measures in the BPFs and along the trade routes are non-existent [3,4]. The movement of live birds between BPFs and transportation to urban/peri-urban LBMs potentially facilitates the spread of viral diseases to naïve poultry [5]. Poor poultry management favors the existence and spread of diseases due to gregarious interactions of different poultry species within BPFs and frequent introductions of birds sourced from LBMs [6]. Recent data suggest that wild birds at BPFs contribute to the circulation of NDV and spill-over of NDV vaccine into wild avian species [7,8].

Newcastle disease (ND) is a serious respiratory and neurological disease and a major challenge for backyard and commercial poultry production [9,10]. The disease is prevalent and consistently reported from all continents, and outbreaks occur worldwide [11]. In 2017-2019, over 20 Asian and 30 African countries reported over 5400 ND outbreaks to the World Organisation for Animal Health (OIE), a large fraction of which were from Iran (*n* = 492), Ghana (*n* = 454), Afghanistan (*n* = 442), and Zambia (*n* = 425); only 40 reported outbreaks were from Kenya [12]. However, ND outbreaks are likely under-reported due to poor surveillance [13].

The causative agent of ND, Newcastle disease virus (NDV), is classified as an avian orthoavulavirus-1 in the family *Paramyxoviridae,* genus *Orthoavulavirus* [14]. Viruses with an intracerebral pathogenicity index (ICPI) score of at least 0.7 in 1-day-old chicks, or having a multibasic amino acid motif at the fusion protein cleavage site (demonstrated directly or by deduction) are classified as virulent (vNDV) and their presence is reportable to OIE [15]. The single-stranded, negative-sense, non-segmented 15.2 kb genome (i.e., one of three genome sizes: 15,186, 15,192, and 15198 nucleotides) of NDV encodes six proteins: nucleoprotein (NP), phosphoprotein (P), matrix (M), fusion (F), hemagglutinin-neuraminidase (HN), and polymerase (L); two additional proteins (V and W) are produced by RNA editing during transcription of the P gene [9,16]. Based on virulence in chickens, NDVs are categorized as velogenic (induce high morbidity and mortality), mesogenic (mainly induce respiratory disease, occasional nervous signs, and low mortality), lentogenic (induce subclinical to mild respiratory disease in younger chicks), and asymptomatic (subclinical enteric infection) [11,17]. Based on the disease induced in chickens under laboratory conditions, velogenic NDVs are further descriptively classified into viscerotropic (cause severe hemorrhagic intestinal lesions and up to 100% mortality) and neurotropic (induce neurologic signs and about 50% mortality) [18]. NDV isolates cluster phylogenetically into class I (mostly of low virulence; predominantly in wild birds) and the highly diverse class II (with genotypes designated I-XXI) [19].

Genotype V (formerly Vd) has been associated with ND outbreaks worldwide. Disease in psittacines in South America and their importation to Europe and the USA in the early 1970s has been linked to the spread of these viruses [20,21]. Some reports have documented the simultaneous emergence and spread of genotype XII viruses from the Far East [20]. The significant genetic diversity of the genotype V viruses led to the identification of subgenotypes V.1 (formerly Vb) and V.2 (formerly Vc). In addition, three sub-groups of diverse viruses have been previously assigned to the lower order (i.e. V) as they did not meet all the criteria for classification into separate sub-genotype [19]. These groups represented viruses from Europe (1970s–1980s), South America (1975–2006), and East Africa (Uganda in 2011, Tanzania in 2012, and Kenya during 2010–2016). The sub-group from East Africa lacked at least four independent isolates without a direct epidemiologic link and bootstrap value of at least 70%, which are required for the assignment of NDVs to a new sub-genotype [19]. Subgenotypes V.1 and V.2 viruses are viscerotropic velogenic, with V.1 viruses prevalent in commercial birds in some countries in the Americas [19,22,23]. Other genotype V viruses have in the recent past been detected in the East African region [24,25,26]. There is a scarcity of data on the NDV genotypes circulating among Kenyan poultry in LBMs and BPFs.

Here, a surveillance study was implemented to investigate whether vNDVs were detectable in both healthy birds and birds showing clinical signs consistent with ND from different ecogeographical and socio-cultural regions in Kenya. The aim of the study was to identify and characterize NDV variants that affect domestic avian species in Kenya and thus contribute to disease surveillance and reporting in the country. This surveillance involved collection of oral and cloacal swabs from indigenous chickens, wild farm birds (WFBs; i.e., wild birds frequently found at the BPFs), and other domestic poultry species at BPFs and LBMs, and testing for virus presence by real-time reverse transcription-polymerase chain reaction (rRT-PCR). Virus isolation from a subset of samples in specific pathogen-free embryonated chicken eggs was attempted and analyzed by random next-generation sequencing (NGS). A time-scaled phylogeographic analysis was performed to model the spatial-temporal dispersal patterns of the viruses.

## 2. Materials and Methods

### 2.1. Sampling Sites

**Figure 1:**
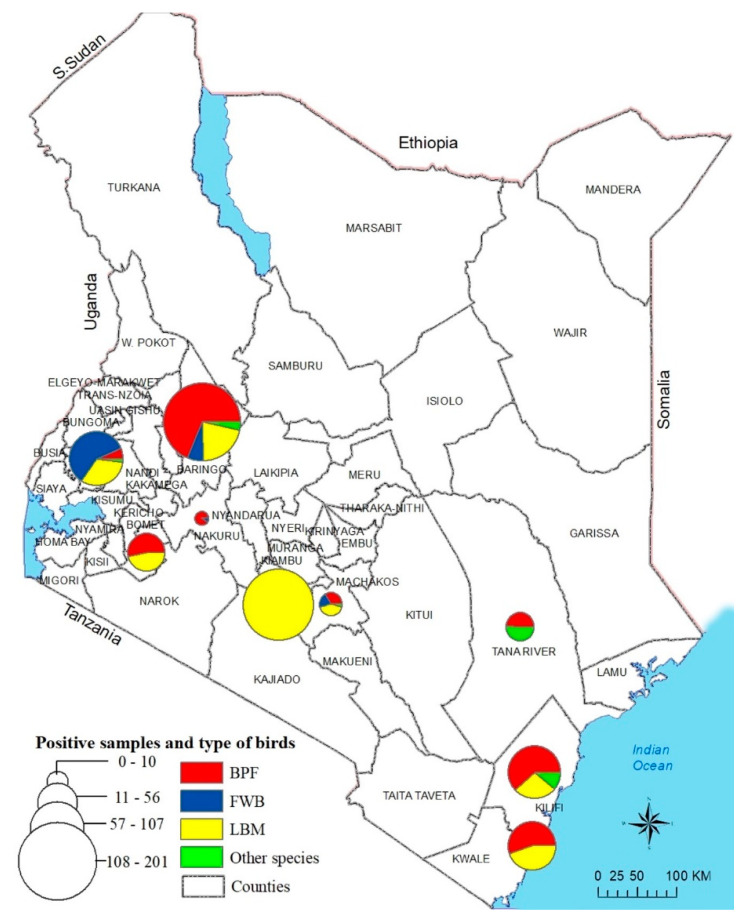
Sampling sites in nine counties of Kenya from where the NDV-positive samples originated. The sampling sites were mapped using GPS coordinates recorded during the sampling. A total of 641 birds swabbed from 66 BPFs and 21 LBMs from nine counties in Kenya tested positive for NDV by rRT-PCR using the L-tests described in the text. Circle sizes correspond to the numbers of virus-positive samples. Abbreviations: BPF, backyard poultry farm; WFB, wild fam birds.

Clinical samples from poultry and non-poultry species were collected in 2017–2018 at 66 rural BPFs and 21 urban/peri-urban LBMs in nine Kenyan counties (Table S1). The BPFs were in Baringo (*n* = 11), Bomet (*n* = 2), Tana-River (*n* = 6), Kakamega (*n* = 6), Kilifi (*n* = 19), Machakos (*n* = 9), Mombasa (*n*=9), and Nakuru (*n*=4) counties, while the LBM were in Baringo (*n* = 4), Bomet (*n* = 1), Kakamega (*n* = 3), Kilifi (*n* = 1), Machakos (*n* = 2), Mombasa (*n* = 2), and Nairobi ( *n*= 8) counties. A description of the Kenyan LBMs is provided in Table S1. Four of the 21 LBMs were open-air markets (i.e. Mogotio, Riwo, Kapkwen, and MwembeKuku), 16 LBMs were indoor markets, and one LBM (Kipkaren) had one weekly open-air market day and a daily indoor market. Birds sold at the indoor LBMs were housed throughout in wire-mesh cages (8–20 birds per cage). The weekly turnover at the LBMs averaged from 200 to 1000 birds per week depending on the type of market. Counties with high densities of free-range indigenous chickens (Kakamega and Machakos) and large water bodies, such as lakes that attract migrant species (Baringo and Nakuru), were selected for the sampling of WFBs. All field-work activities were conducted in collaboration with the regional Central Veterinary Laboratories (CVLs) (Figure 1).

### 2.2. Sample Collection

Samples were collected from indigenous chickens, WFBs, and other domestic poultry species found at BPFs and LBMs. From each farmer at the BPFs or trader at the LBMs, at least four birds were randomly selected as representative portions of the poultry flocks. For farmers with multiple floors or houses or traders with multiple stalls/cages, birds were randomly sampled to represent each floor/house/cage. Adult birds at BPFs and birds that had stayed the longest time at LBMs were preferentially swabbed. Targeted WFBs included species in the orders of Columbiformes, Passeriformes, Psittaciformes, Galliformes, and Anseriformes. Sampling data included species of birds, GPS coordinates, sampling dates, observed clinical signs consistent with ND (e.g. depression, respiratory distress, neurological signs), and vaccination histories. The WFBs were captured by mist nets and marked with branded leg rings to prevent re-sampling. From each bird, an oropharyngeal (OP) and a cloacal (CL) swab were collected using sterile, plastic-shafted flocked swabs (Puritan Medical, Guilford, ME). Each swab was placed in individual 2.0 mL Corning® cryogenic vials (Corning Inc., Corning, NY, USA) containing 1.5 mL of viral transport media (brain-heart-infusion broth; Difco, NZ) supplemented with antibiotics and antifungals according to standard procedures [27]. Swabs were immediately stored in liquid nitrogen and preserved at –80℃ until shipment to Southeast Poultry Research Laboratory (SEPRL) of the ARS-USDA in Athens, GA, USA for analyses.

### 2.3. Total RNA Extraction and Virus Detection

Total RNA was extracted from 50 μL of each CL or OP sample (prepared separately for each bird) by MagMAX™-96 AI/ND Viral RNA Isolation Kit (Ambion, Inc.) on an automated KingFisher Magnetic Particle Processor (Thermo Fisher Scientific, Waltham, MA, USA). All real-time reverse transcription-polymerase chain reaction (rRT-PCR) tests were performed using AgPath-ID™ one-step RT-PCR kit (Ambion Inc., Austin, TX, USA) on an ABI 7500 Fast Real-Time PCR System (Applied Biosystems). Samples with cycle threshold (*C*_T_) values of less than 40 were considered as putative positives.

Samples were tested by rRT-PCR targeting the NDV large polymerase gene (L-test) using the forward primer L+12170 (5’-ACA GCT GGG AAT CTC CAA CA-3’), reverse primer L-12282 (5’-CTT TGA GAA TCA TTG GAT ATG TGA-3’), and probe L+12212 (5’-CAG ATG ACA TTT ACC CCT GCA TCT CT -3’) as previously described [28]. The L-test was performed in 25 µL reaction volumes comprised of 8µl of total RNA, 12.5 µL of (2×) buffer, 0.5 µL of the forward and reverse primers (20 pmol/µL), 0.5 µL of the probe (6 pmol/µl), 1 µL of AgPath enzyme mix (25×), and sterile nuclease-free water. The test included an initial RT step (10 min at 45 °C and 10 min 95 °C), and PCR steps of 40 cycles (10 s at 95°C, 30 s at 57°C, and 10 s at 72 °C). Samples that had *C*_T_ values of ≤30 by the rRT-PCR L- test were further tested using established fusion (F-) test primers [29], but with a modified probe F+4894 (5’-[FAM]-AAG CGT TTC TGT CTC CTT CCT CCA-[BHQ]-3’) and amplification conditions (annealing at 58°C) for detection of virulent NDVs in the Kenyan samples.

Relationships between the NDV-positive samples (by the L- and F-tests) and the sampling areas (counties) were established using customized ggplot2 scripts in RStudio v 1.2.5033 (RStudio Inc., Boston, MA, USA) (http://www.rstudio.com/).

### 2.4. Virus Isolation

Samples with *C*_T_ values ≤30 by the rRT-PCR L-test and positive by the F-test were selected for virus isolation in 9-to-11-day-old specific pathogen-free embryonated chicken eggs (SPF-ECEs) (3 eggs per sample; up to 2 blind passages). The inoculated SPF-ECEs were incubated for a maximum of 7 days. Allantoic fluids were then harvested from the SPF-ECEs with embryo mortality on the day of mortality as well as those that were alive at the end of the 7-day incubation period. This fluid was then tested by hemagglutination assay (HA) using standard procedures [30]. Total RNA was extracted from 250 µL of the harvested hemagglutinating allantoic fluids using Invitrogen™ TRIzol® LS/Viral RNA Mini Kit (Qiagen Inc, Germantowm, MD, USA) following the manufacturer’s instructions. The extracted total RNA was used to detect NDV by rRT-PCR in the allantoic fluid as described above. Intracerebral pathogenicity index (ICPI) was conducted on one isolate to assess virulence using 1-day old white leghorn chickens per OIE recommendations [30].

### 2.5. Next-Generation Sequencing

All the samples with *C*_T_ values of ≤30 by the rRT-PCR L- test were used for the total RNAs extracted from swabs and allantoic fluids were used to generate DNA sequencing libraries (one library per sample) using the KAPA Stranded RNA-Seq Kit (Roche, Inc., Branchburg, NJ, USA). Libraries were then purified with Agencourt® AMPure®XP beads (Beckman Coulter, Brea, CA, USA) and quantified using Agilent High Sensitivity DNA Kit on an Agilent 2100 Bioanalyzer (Agilent Technologies Inc., Santa Clara, CA, USA). For sequencing, the libraries were diluted to 4 nM final concentration, and then equimolar volumes (10 µL of each library) were pooled, briefly vortexed, and incubated for 5 min at room temperature. The pooled libraries were further diluted to a final concentration of 10 pM, followed by the addition of 3% PhiX control library (Illumina, San Diego, CA, USA). Paired-end sequencing (2 × 250 bp) of the pooled libraries was performed on an Illumina MiSeq platform using the 500 cycle MiSeq Reagent Kit v 2 (Illumina, San Diego, CA, USA). Raw sequence reads were analyzed and assembled by MIRA v 3.4.1 [31] within customized workflows on the Galaxy platform [32,33].

### 2.6. Genome Sequence Assembly and Characterization

#### 2.6.1. Sequence Assembly and Phylogenetic Analyses

Sequencing reads obtained from the OP and CL MiSeq data were merged for each bird and *de novo* assembled, and the assembled contigs annotated with the lowest common ancestor based on BLASTn algorithm [34] executed on a Geneious Prime 2020.1.2 platform [35]. Consensus sequences of the complete genomes and the fusion genes of the NDV isolates were compared with representative isolates retrieved from the GenBank; 49 complete genome and 56 fusion gene sequences of viruses in genotypes II, V, and VI were retrieved. Multiple sequence alignment was performed using MUSCLE v 3.8.425 [36] and used to construct phylogenetic trees by maximum likelihood method based on the GTR model of nucleotide substitution with gamma-distributed rate variation among sites and bootstrap resampling (1000 replicates) in MEGA X [37]. The recently revised NDV classification criteria for genotype and sub-genotype identification was followed in this study [19]. Fusion gene cleavage site motif was evaluated for the presence of multiple basic amino acid residues at the C-terminus of the F_2_ protein (at least three arginine or lysine residues between positions 113 and 116) and a phenylalanine residue at the N-terminus (position 117) of the F_1_ protein according to OIE code [15]. The antigenic sites of the attachment (HN) glycoprotein [38] were analyzed for the presence of amino acid substitutions.

The complete or nearly complete genome sequences of the 32 Kenyan NDV isolates identified in the current study have been deposited in GenBank with accession number MW342776-MW342807.

#### 2.6.2. Bayesian Evolutionary and Spatial-Temporal Dispersal Inferences

Time-scaled phylogeographic relationships between the NDV isolates (based on F-gene) were inferred using BEAST v 1.10.4 with the BEAGLE library [39]. For BEAST, an XML data file was created by configuring a MEGA nucleotide alignment file and a taxa traits file (sampling dates/GPS data) in BEAUti v 1.10.4. Evolutionary rate variations and population dynamics among the taxa sequences were estimated using an HKY nucleotide substitution model (Gamma+4 distribution; “(1st+2nd)+3rd” codon positions) with a Bayesian SkyGrid population model and an uncorrected log-normal distribution model under a relaxed molecular clock model [40,41]. Sampling and convergence results of the BEAST model parameters were examined using Tracer v 1.7.1 [42] and the maximum clade credibility (MCC) trees were annotated using TreeAnnotator v 1.10.4 [39]. Annotated trees were visualized and rendered using FigTree v 1.4.4 [43] and the resultant well-supported rates of evolutionary dispersal of the isolates were identified and rendered using SreadD3 v 0.9.6 through a Bayes factor test [44].

## 3. Results

### 3.1. Sample Analyses

#### 3.1.1. Sample Collection

Overall, 1621 birds (one CL and one OP swab per bird; total of 3242) sampled at 66 BPFs and 21 LBMs in Baringo (*n* = 269), Bomet (*n* = 127), Tana-River (*n* = 112), Kakamega (*n* = 302), Kilifi (*n* = 240), Machakos (*n* = 181), Mombasa (*n* = 118), Nairobi (*n* = 177), and Nakuru (*n* = 95) were tested by the rRT-PCR tests (Table 1). Most of the samples were swabbed from chickens at the BPFs (*n* = 914) and LBMs (*n* = 431). All the LBM birds were chickens; there were no LBM birds sampled from Tana-River and Nakuru counties, and all birds from Nairobi county were swabbed at LBMs (*n* = 8).

Samples from WFBs (*n* = 276) included African citril, weavers (baglafecht weaver, black-headed weaver, and white-browed sparrow-weaver), bronze manikin, common bulbul, doves (emerald-spotted wood dove, laughing dove, and ring-necked dove), sparrows (grey-headed sparrow, house sparrow, and Kenyan rufous sparrow), red-billed fire-finch, superb starling, flycatchers (white-eyed slaty flycatcher, blue-mantled crested flycatcher, and northern black flycatcher), and red-cheeked cordon-bleu. Samples from other domestic poultry species (*n* = 111) included duck, goose, guinea fowl, pigeon, quail, and turkey.

#### 3.1.2. Vaccination Histories and Clinical Signs Consistent with ND

During sampling, it was noted that vaccination was not consistently applied across the different counties in Kenya. In fact, some of the farmers at the BPFs whose flocks had purportedly been vaccinated did not keep any vaccination records; most farmers were unaware of the existence of government-sponsored vaccination programs. A large presence of non-vaccinated birds was always present across all the sampled geographical regions. Only chickens swabbed at BPFs in four locations in Kilifi county (Sokoni, Mtwapa, Rabai, and MwembeKuku) and at Changamwe LBM in Mombasa county showed clinical signs consistent with ND. The clinical signs noted in chickens in Sokoni were in two neighboring BPFs and had started three days prior to the sampling date, followed by sudden deaths of more than 25% of the flocks within two days after sampling. The flocks in these two BPFs had been vaccinated less than 3 months prior to the sampling dates. The flocks sampled from all the other sampling locations were apparently healthy at the time of sampling.

### 3.2. Virus Detection

The L-test detected NDV nucleic acids (cut off *C*_T_ < 40) in 39.5% (*n* = 641) of the 1621 tested samples from the chickens, WFBs, and other domestic poultry species obtained from 87 locations across the nine Kenyan counties (Figure 1 and Table S1).

The prevalence of NDV-positive samples was twice as high in chickens sampled at the LBMs (71.7%; 309/430) than the chickens from the BPFs (31.3%; 286/914). The virus was detectable only in 10.9% (18/165) and 25.2% (28/111) of the WFBs and other avian species, respectively, all with high *C*_T_ values (35.6–37.1, and 32.7–38.0, respectively).

**Figure 2:**
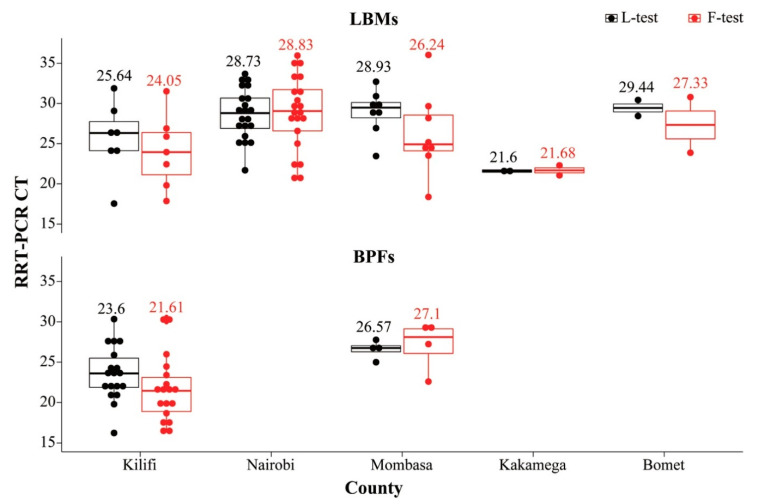
Plot of 62 indigenous chickens sampled from LBMs and BPFs in five Kenyan counties that were confirmed as NDV-positive by the rRT-PCR L- and F-test (in black and red, respectively). The mean rRT-PCR *C*_T_ values obtained from each of the two tests are shown. Note that there were no BPF birds from Nairobi county, and all the BPF birds sampled from Bomet county had *C*_T_ values above 30.

Of the above-mentioned 641 samples, 70 samples that had *C*_T_ ≤ 30 were further tested by the F-test to determine if they were likely virulent virus. The F-test confirmed 62 of the samples as NDV-positive with *C*_T_ values ranging from 16.28 to 36.03; most of these samples (*n* = 53) had *C*_T_ ≤ 30. All 62 samples were from birds sampled from Nairobi (21 LBM indigenous chickens), Mombasa (4 BPF; 8 LBM birds), Kilifi (18 BPF; 7 LBM birds), and two LBM birds each from Kakamega and Bomet counties. Figure 2 shows the distribution of the 62 NDV-positive samples (as confirmed by the L- and F-tests) across the five counties, with samples from Kilifi BPFs and Kakamega LBMs having the lowest mean average *C_T_* values (~21 to 23) for both tests.

### 3.3. Characteristics of the Identified Kenyan NDV Isolates

The virus was isolated in SPF-ECEs from 44 of the 55 samples that tested positive by the F-test, of which 28 samples were from LBMs in Nairobi (*n* = 12), Kilifi (*n* = 7), Mombasa (*n* = 6), Kakamega (*n* = 2), and Bomet (*n* = 1), while 16 samples were from BPFs in Kilifi (*n* = 12), Kakamega (*n* = 3), and Mombasa (*n* = 1) counties. The virus was sequenced by NGS from both the harvested allantoic fluids and their corresponding original swabs, which resulted in 32 complete genome sequences of NDV isolates (Table 2). Twelve of the isolates were from samples collected at BPFs in Kilifi; the remaining 20 were from LBMs in Nairobi (*n* = 13), Mombasa (*n* = 4), Kilifi (*n* = 2), and Bomet (*n* = 1).

#### 3.3.1. Genomic Characteristics

Sequence analyses showed that of the 32 Kenyan NDV isolates, 30 isolates had consensus genome sequence lengths of 15,192 nucleotides (nt), except one isolate (A142) which had two gaps (missing bases 1500–1534 and 1605–1648). The other two isolates (34MB09and 1MB35) were missing seven bases at either the 5’ or 3’ ends and with a consensus sequence length of 15,185 nt. The %GC content of the genomes ranged from 46.1% to 47.7%. The genome sequences of all 32 isolates were supported by mean coverage of 99.97–100%, and all but one isolate had a median read depth ranging from 1000 to above 10,000 (Table 2). Isolate A369 was the lone exception with a low number of mapped reads (*n* = 2275) and median read depth (13x); further analyses of the NGS reads from the sample containing the isolate A369 revealed that most of the reads (*n* = 516,877) mapped to influenza A virus H9N2, which has been recently published (GenBank accession numbers MN242734 to MN242774) [45].

The F-gene nucleotide sequence identities amongst the 32 Kenyan NDVs was 95.8–100% (Figure S1); the 95.8% identity was between sequences from Kangemi (A5136/17) and Burma (A142/17) LBMs and the viruses from Kilifi BPFs (3FB34/18, 3FB/18 and 3FB/18). The Kenyan nucleotide sequence had identities of 95.6–98.2% to genotype V sequences detected in East-Africa since 2010; the lowest was between a 2016 isolate (MG988405/ck/Kenya/Amagoro/KE1007/16) and several sequences from Kilifi BPF. The 2016 isolate also showed the highest identity (98.2%) with A5136/17 and A142/17 sequences from Nairobi LBMs. Comparing the new Kenyan and V.1 nucleotide sequences, MK040382/ck/CA/B1800621-1.8/18 was 86.5% identical to A5136/17 and A142/17, while the sequence of A144/17 from Burma LBM in Nairobi was 88.9% identical to both JN872194/ck/Honduras/498109-15/07 and AY562987/gamefowl/U.S.(CA)/211472/02). Genotype V sequence identities with Kenyan sequences was 88.5–91.9%; MK046920/ck/Jalisco/LVVX17186/2017 was the least identical (to 3FB34/18, 3FB128, and 3FB35 from BPF sequences), while KJ577136/ck/Chimalhuacan/1973 was the most identical (to A375/17 LBM sequence from Nairobi).

Comparison of the deduced F protein sequences (553 amino acid residues) of the 32 Kenyan NDV isolates with published sequences revealed that the F_0_ precursor cleavage site of all 32 Kenyan isolates contained three basic amino acid residues at the C-terminus of the F_2_ protein and a phenylalanine (F) residue at the N-terminus of the F_1_ protein (^113^R-Q-K-R↓F ^117^), which typically corresponds to a virulent phenotype. Further, the HN glycoprotein (571 amino acid residues) of all 32 Kenyan NDV isolates revealed a conserved sialic acid binding site (NRKSCS; position 234–239), 13 conserved cysteine residues (positions 123, 172, 186, 196, 238, 247, 251, 344, 455, 461, 465, 531, and 542), and three key receptor-binding amino acid residues at positions 401 (E), 416 (R), and 526 (Y) [38,46]. The antigenic sites of the HN glycoprotein contained a total of five amino acid residue substitutions at position 201 (H to Q) in two isolates from LBMs in Nairobi (A375) and Kilifi (3MB14); positions 343 (T to S) and 521 (S to N) in two Nairobi LBM isolates (A142 and A5136); position 347 (E to K) in four of the 2018 isolates from Changamwe LBM in Mombasa (1MB23, 1MB35, 1MB40, and 1MB42); and position 494 (N to D) in isolate 3MB14 (Figure S2). Another amino acid substitution was observed in the fusion promotion region at 145 (I to M) in six isolates (A101, (A104, (A105, 3MB09, 3FB128, and 3FB129).

#### 3.3.2. Phylogenetic and Phylogeographic Analyses

**Figure 3:**
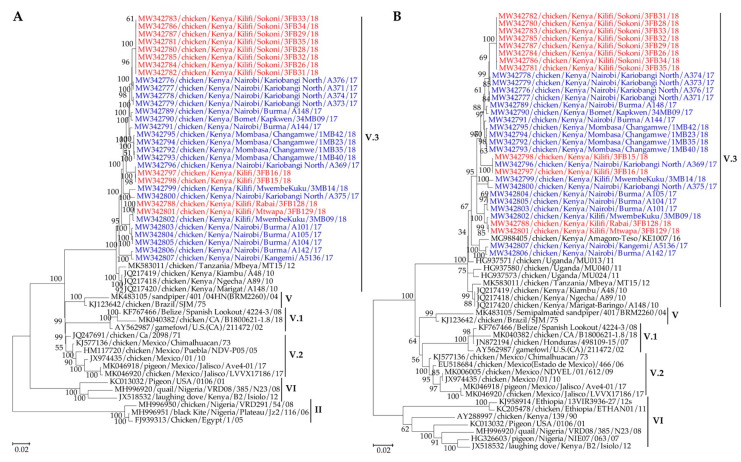
(**A**) Phylogeny of the complete genome nucleotide sequences and (**B**) fusion gene nucleotide sequences of 32 NDV isolates reported in the current study in comparison to related isolates in GenBank. The sequences of the 20 and 12 NDV isolates isolated from the LBMs and BPFs are shown in blue and red fonts, respectively. The evolutionary history was inferred by using the ML method based on the GTR model [47]. The analysis involved 53 and 59 nucleotide sequences for the complete genomes and the fusion genes, respectively. Codon positions included were 1st+2nd+3rd+Noncoding. All positions with less than 95% site coverage were eliminated, i.e., fewer than 5% alignment gaps, missing data, and ambiguous bases were allowed at any position (partial deletion option). The final datasets had 14795 and 1662 positions for the complete genomes and the fusion genes, respectively. Evolutionary analyses were conducted in MEGA X [37].

Phylogenetically (based on complete genome, F and HN gene nucleotide sequences), the Kenyan isolates clustered together with other east African isolates into a distinct sub-clade that was different from isolates from subgenotypes V.1 (from USA and Belize) as well as V and V.2 (from South America) (Figure 3 and Figure S2A).

Based on the distinct ecogeographical origins, and strong bootstrap support values (70–100%), the Kenyan NDVs could be classified into a new subgenotype V.3, with sub-clustering depending on the sampling location and type of the sampled birds (BPFs vs LBMs). For instance, nine isolates obtained at BPFs in Sokoni (Kilifi county) clustered together in a distinct subclade. The same pattern was apparent in the clustering of the isolates obtained from birds sampled at Kariobangi North and Burma LBMs in Nairobi and Changamwe LBM in Mombasa. A phylogenetic analysis based on the full-length nucleotide sequence of the fusion gene of the 32 Kenyan subgenotypes V.3 viruses and selected isolates representing all NDV sub/genotypes available in the NDV consortium repository [19] is shown in Figure S3.

When the full-length F-gene nucleotide sequences were used to estimate the genetic distances between the viruses in subgenotypes V.1, V.2, and V.3, the average distance per site ranged from 0.000 to 0.044 (Table S2). Based on the current NDV classification system, the new V.3 viruses could not be further delineated as the average distance per site should be above 5% (0.05) [19]. The most divergent virus from the V.3 sequences presented in the current study was the 2016 isolates (MG988405/ck/Kenya/Amagoro/KE1007/16)from Amagoro county in Western Kenya, followed by the 2012 Tanzanian isolates (MK583011/ck/Tanzania/Mbeya/MT15/12), then the 2010–2011 Ugandan isolates, and the 2010 isolates from Kiambu county in central Kenya. From the data presented in Table S2, the genetic distances amongst the V.3 viruses appear to be dependent on the locations (counties) and type (BPFs vs LBMs).

**Figure 4:**
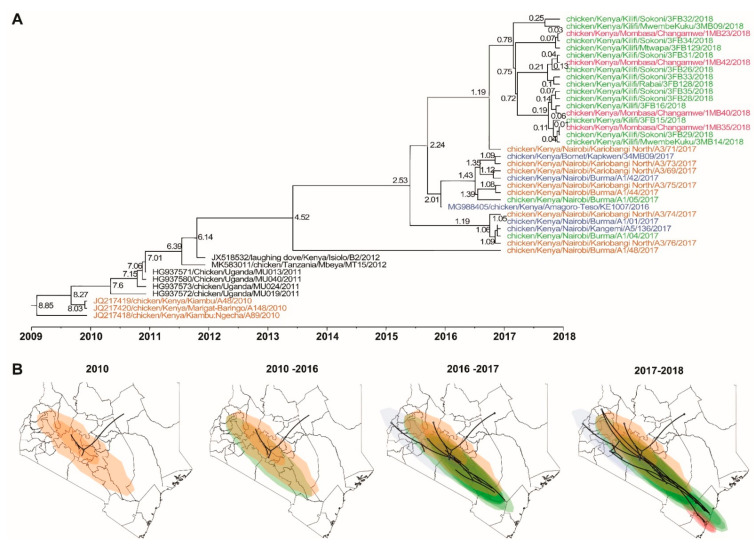
(**A**) Time-scaled phylogeographic relationships between the NDV isolates in east African region, and (**B**) a west-to-east spatial-temporal dispersal of the NDV genotype V.3 isolates between 2010 and 2018 in Kenya. The analyses were performed based on the nucleotide sequences of the NDV F-gene and included the 32 Kenyan isolates sequenced in the current study and previously sequenced isolates reported between 2010 and 2016. The numbers on the phylogeographic tree nodes (panel **A**) represent tip height (from the oldest to the youngest isolate). Note that the isolates are color-coded depending on the sources of the chickens from which they were isolated (see a magnified map in Figure S4).

A time-scaled phylogeographic analysis (based on F gene nucleotide sequences) suggests multiple potential sources of the Kenyan NDVs, including chickens from western Baringo county and Kiambu (a Nairobi cosmopolitan township) in 2010. The 2017 isolates from Nairobi LBMs were from chickens sourced from western Kenya in Busia, Baringo, Trans-Nzoia, and Bomet counties (highlighted in orange and blue in Figure 4).

Some of the 2017 isolates identified from the Burma market in Nairobi and the 2018 isolates from Kilifi and Mombasa counties were isolated from chickens originating from the Eastern region (Machakos and Makueni counties) (highlighted in green in Figure 4). All four 2018 isolates from Changamwe LBM in Mombasa county were from birds sourced from BPFs within the county (highlighted in pink in Figure 4). Details of the spatial-temporal dispersal of the NDVs are presented in Figure S4. Although the virus origins could not be confirmed due to the scarcity of continuous and systemic surveillance data in Kenya, the data suggest a west-to-east dispersal of the NDVs, most likely through the live chicken trade.

#### 3.3.3. Biological Characteristics

One of the 2018 isolates from Sokoni BPF in Kilifi (isolate 3FB31) was selected for ICPI testing and had a value of 1.86 (with a maximum of 2.0), which is above the ICPI minimum threshold value of 0.7 indicative of highly virulent NDV. This sample had low rRT-PCR *C*_T_ values (19.78 and 20.22 by the L- and F-tests, respectively) and had been obtained from a vaccinated flock that also showed clinical signs consistent with ND. Seven other 2018 isolates (3FB26, 3FB28, 3FB29, 3FB32, 3FB33, 3FB34, and 3FB35) from Sokoni were from purportedly vaccinated flocks that also presented clinical signs at the time of sampling, all of which had *C*_T_ < 25 by both the L- and F-tests (Table 2). Other isolates from nonvaccinated but sick birds were from Kilifi town (3FB15 and 3FB16), MwembeKuku (3MB09), Rabai (3FB128), Mtwapa (3FB129), and Mombasa (1MB23, 1MB35, 1MB40, and 1MB42). The three birds swabbed from Mombasa were from one trader from Changamwe LBM. The traders from Changamwe LBM, who are usually supplied with chickens from rural farmers from the neighboring Kilifi counties, reported frequent outbreaks in the market. In summary, only 17 out of the 32 isolates reported here were from birds that presented ND-like clinical signs; flocks from which the remaining 15 isolates were derived were apparently healthy at the time of sampling.

## 4. Discussion

This study presents data on the surveillance, genotyping, and dispersal patterns of NDVs in Kenyan backyard poultry farming and in the birds traded at the markets. Assessment of the NDV distribution is important considering the absence of continuous and systemic disease surveillance in Kenya. Notably, only farmers near the sparse CVLs report ND outbreaks, and to avoid incurring losses, most farmers sell off their flocks to unsuspecting neighbors or local middlemen upon noticing the first signs of infections [48].

The genomes of all 32 identified NDVs conformed to the “rule-of-six” indicative of efficient virus replication [49]. Further, the confirmatory F-test, high ICPI score, and F-gene cleavage pattern show that the viruses are highly virulent. The current study has classified the Kenyan vNDVs into a new subgenotype V.3, which, in accordance with the recently revised NDV classification system [19], is supported by the presence of several independent isolates without a direct epidemiologic link and phylogenetic clustering with bootstrap values of more than 70%. The Kenyan vNDV V.3 isolates are closely related to four other East African isolates identified with complete genome sequences [25,50], and others identified with partial F and HN gene sequences from Uganda in 2011 [24] and Kenya in 2018 [26]. So far, no other NDV strains related to subgenotype V.3 viruses have been reported from Northern, Western, and Southern Africa, suggesting that the viruses may be geographically restricted to the East African region. Except for South Africa and part of Western Africa (e.g., in Nigeria), NDV genetic diversity and epidemiology are poorly described in Africa. Congruency in phylogenetic clustering, and the various amino acid substitutions observed at antigenic/neutralizing regions of the HN glycoprotein, supports the V.3 classification. Although the genetic distances of the V.3 viruses were less than 5% among all Eastern African sequences, they were higher when compared to the viruses detected in 2010–2012 than those detected in 2015-2018. This finding suggests a local diversification of the viruses in the East African region, as well as specific locations (e.g., rural farms vs the markets). Previous studies using a panel of neutralizing anti-HN monoclonal antibodies have described seven overlapping antigenic sites (designated as sites 1, 2, 3, 4, 12, 14, and 23) whose amino acid substitutions could lead to conformational and antigenic changes and thus alter the functional characteristics of the HN glycoprotein [51]. Such antigenic changes could potentially affect the virus attachment, assembly, and maturation processes that are mediated by the HN glycoprotein [52]. Therefore, mutations in the HN epitope regions, coupled with suboptimal vaccinations and other genetic modifications, could undermine the management of ND.

The finding of higher NDV prevalence amongst the chickens at the LBMs compared to BPFs is important because LBMs are the backbone of the Kenyan poultry industry; 20 of the 32 vNDV isolates described here were from LBM chickens. In Kenya, there is no virus screening along the dominantly informal and unregulated (i.e., without any biocontrol measures) chicken marketing chains, which have recently been reported to result in an increased frequency of ND outbreaks in the country [3]. Further, depending on factors such as price fluctuations, the anticipation of holidays and festivals, etc., many Kenyan traders opt to keep their birds for extended periods of time during which new birds from various localities are introduced and mixed with old stocks in unhygienic and crowded cages, often with insufficient feed/water or veterinary services. Further, poultry middlemen and traders collect birds and transport them by any available means (public buses and private trucks) with or without cages to various markets depending on volumes of weekly trade. For instance, the birds sold at LBMs in Nairobi, which had the highest virus prevalence in the current study, originated from at least 10 different counties spanning the Western, Rift Valley, Central and Eastern regions of Kenya. The Western region, which has the highest population of small-scale domestic poultry in Kenya, is particularly important as it serves as the chicken source to many urban markets within the country, as well as across borders to Uganda (Busia, Bungoma, and Trans-Nzoia counties) and Tanzania (Narok and Bomet counties). Equally important is the Eastern region, especially Makueni and Machakos counties, which supply chickens to Nairobi markets (adult birds for slaughter mainly carried out at the markets) and the coastal region (young chicks for rearing at rural BPFs and subsequent sale at the markets). The phylogeographic analyses suggest a west-to-east virus dispersal, most likely through live chicken trade and markets. Since NDV is primarily transmitted via aerosols or ingestion of virus shed in feces and respiratory secretions originating from infected birds [53], live chicken trade and markets likely provide conducive environments for the generation of, and sustainable reservoirs for the maintenance and dissemination of, new virus variants.

Since vaccination is an important biosecurity measure against ND [33], the detection and isolation of NDVs from purportedly vaccinated flocks in the current study raises issues such as the possible presence of live vaccine viruses in the sampled flocks. Another issue is mismanagement of vaccination regimes (e.g., use of a vaccine that has been inactivated or incorrect application of vaccines), which could result in failure to stimulate protective immune responses and prevent replication of virulent viruses. The most widely available vaccine in Kenya is an avirulent thermostable I-2 live-attenuated vaccine (supplied from Australia as a seed strain to produce ND vaccines for rural village poultry [54]) and is administered by eye drops in chickens of all ages. However, this vaccine is not accessible to most rural farmers in Kenya [48], and although it is more thermostable than other ND vaccines, the vaccine cold chain must still be maintained [55,56]. The lack of cold chains in remote rural areas could compromise its efficacy. Further, the subgenotype V.3 NDVs circulating amongst the Kenyan rural poultry populations is phylogenetically divergent from the vaccine used, implying potentially reduced ability of the vaccines to prevent the shedding and continuous maintenance of vNDVs in the environment [57,58]. It should be noted that the rRT-PCR F-test used in the current study is targeted to the F gene cleavage site to differentiate vNDVs from low virulent viruses and would not identify vaccine viruses. Another important biosecurity measure against ND is the depopulation of infected or exposed chickens and restrictions in poultry movements, especially during outbreaks. Kenya lacks government-sponsored programs for culling and compensation to farmers for the loss of their flocks to ND. This, combined with improper disposal of sick chicken carcasses, may facilitate NDV spread.

Finally, the current study points to the presence of NDVs in other domestic poultry species (duck, goose, guinea fowl, pigeon, quail, and turkey) and WFBs that gregariously mingle with domestic chickens. Numerous L-test rRT-PCR positives with high C*_T_* values were identified in birds other than chickens, but because of our testing protocols, we did not confirm by sequence or isolation if this virus was spillover from the domestic chickens sampled in particular BPFs, or belonged to genotypes other than the vNDV subgenotype V.3 detected in the analyzed chicken samples. It should also be noted that most of the WFBs that tested positive for NDV were from the Western Kenyan sampling sites, which could be attributed to more WFBs sampled from this region compared to other regions. Nevertheless, the free-range nature of the Kenyan rural poultry at the BPFs allows the chickens to interact with many other birds, including wild, synanthropic avian species that may exploit food and water set out for chickens. Further, the rearing of these other domestic poultry species together with domestic chickens has increased on rural farms, but recent data has shown that farmers that keep these species do not usually report suspected cases of ND to the CVLs [48]. Thus, based on the high NDV prevalence reported here, these bird species could be biological vectors and have the potential to spread the virus in domestic poultry through feeders, drinkers, cages, or feces. There is a need for the characterization of viruses in WFBs and other domestic poultry species, which was not performed in the current study.

## 5. Conclusions

This study identified 32 vNDV isolates from indigenous chickens sampled at Kenyan BPFs and LBMs and has phylogenetically classified the isolates into a new subgenotype V.3 based on the recently revised nomenclature systems of APMV-1. The study has reiterated the endemicity and widespread distribution of vNDVs in Kenya, not only amongst the village backyard chickens but also in other poultry species that are kept together with the village chickens in the BPFs as well as in WFBs that frequent the BPFs. The detection of significantly higher prevalence of NDV at LBMs compared to BPFs, coupled with the phylogenetic clustering of the isolates obtained from the LBMs into separate clades, suggests the possibility of the live chicken trade and markets as foci of uninterrupted replication, sustained maintenance, and dissemination of vNDV variants. The data also affirms the need to establish government-sponsored active surveillance, enforce biosecurity measures at the LBMs, optimize vaccination regimes, and foster general awareness of poultry viral infections amongst the rural farmers who keep over 80% of the chicken population in the country. Given the widespread distribution of vNDVs shown here across the East African region, the need for concerted efforts for surveillance and characterization of viral agents in poultry and determination of their transmission dynamics cannot be overemphasized.

## Figures and Tables

**Table 1:** Sources, number, and types of bird species collected at backyard poultry farms (BPFs) and live bird markets (LBMs) in nine Kenyan counties and considered as “putative Newcastle disease virus (NDV)-positive” based on the detection of NDV nucleic acids by the RRT-PCR L-test (cut off *C*_T_ < 40).

County	Number of Sampling Locations	BPF Chickens		LBM Chickens		Other Domestic Poultry Species ^a^		WFBs ^b^		Total Birds Tested		PCR-Negative Samples
		Total Tested	Putative NDV-Positive	Total Tested	Putative NDV-Positive	Total Tested	Putative NDV-Positive	Total Tested	Putative NDV-Positive	Total Tested	Putative NDV-Positive	
Baringo	13	176	129 (73.3%)	43	41 (95.3%)	7	7 (100%)	43	13 (30.2%)	269	190 (70.6%)	79 (29.4%)
Bomet	4	77	8 (10.4%)	50	18 (36%)	N/A	N/A	N/A	N/A	127	26 (20.5%)	101 (79.5%)
Tana River	6	89	3 (3.4%)	N/A	N/A	23	14 (69.9%)	N/A	N/A	112	17 (15.2%)	95 (84.8%)
Kakamega	8	169	61 (36.1%)	48	35 (72.9%)	32	1 (3.1%)	53	3 (5.7%)	302	100 (33.1%)	202 (66.9%)
Kilifi	20	165	39 (23.6%)	38	9 (23.7%)	37	5 (13.5%)	N/A	N/A	240	53 (22.1%)	187 (77.9%)
Machakos	14	89	5 (5.6%)	30	9 (30%)	12	1 (8.3%)	50	1 (2%)	181	16 (8.8%)	165 (91.2%)
Mombasa	10	74	33 (44.6%)	44	26 (59.1%)	N/A	N/A	N/A	N/A	118	59 (50%)	59 (50%)
Nairobi	8	N/A	N/A	178	171 (96.1%)	N/A	N/A	N/A	N/A	178	171 (96.1%)	7 (3.9%)
Nakuru	4	75	8 (10.7%)	N/A	N/A	N/A	N/A	19	1 (5.3%)	94	9 (9.6%)	85 (90.4%)
Total	87	914	286 (31.3%)	431	309 (71.7%)	111	28 (25.2%)	165	18 (10.9%)	1621	641 (39.5%)	980 (60.5%)

**^a^** other synanthropic avian species: duck, goose, guinea fowl, pigeon, quail, turkey. **^b^** wild farm birds (WFBs): African citril finch, weavers, manikin, common bulbul, doves, sparrows, fire-finch.

**Table 2:** Details of 32 samples from which NDV was successfully isolated and complete genomes obtained by next-generation sequencing (NGS) in the current study.

Collection Date	Isolate	Sampling County (Location; Type)	Samples Used for NGS ^a^	rRT-PCR (*C*_T_)		NGS Assembly Summaries					GenBank Accession No.
				L-Test	F-Test	Mapped Reads ^b^	Coverage Depth ^c^	Consensus Sequence Length (Bases)	Genome Coverage	GC Content	
5/23/2017	34MB09	Bomet (Kapkwen; LBM)	CL (AF)	28.44	23.87	306,471	0|2512|3381|4164|6149	15185 ^d^	99.9%	47.7%	MW342790
3/13/2018	3FB129	Kilifi (Mtwapa; BPF)	CL	22.23	22.26	219,858	0|1971|2588|3126|4500	15,192	100.0%	47.4%	MW342801
3/8/2018	3MB09	Kilifi (MwembeKuku; LBM)	CL	31.88	31.51	107,727	0|1037|1277|1477|2271	15,192	100.0%	47.3%	MW342802
3/8/2018	3MB14	Kilifi (MwembeKuku; LBM)	CL	26.41	22.44	390,144	0|4201|4819|5329|6987	15,192	100.0%	46.3%	MW342799
3/12/2018	3FB128	Kilifi (Rabai; BPF)	CL	22.19	21.83	663,414	0|6280|7810|9291|13482	15,192	100.0%	46.9%	MW342788
3/9/2018	3FB26	Kilifi (Sokoni; BPF)	CL	22.03	17.77	286,241	0|2936|3569|4097|5549	15,192	100.0%	47.2%	MW342784
3/9/2018	3FB28	Kilifi (Sokoni; BPF)	OP	21.81	17.26	311,051	0|2741|3744|4403|6051	15,192	99.9%	46.9%	MW342780
3/9/2018	3FB29	Kilifi (Sokoni; BPF)	CL	24.17	19.73	369,615	0|3659|4273|4757|6271	15,192	99.9%	46.9%	MW342787
3/9/2018	3FB31	Kilifi (Sokoni; BPF)	OP (AF)	19.78	20.22	553,799	2|2488|3999|6750|25170	15,192	100.0%	47.1%	MW342782
3/9/2018	3FB32	Kilifi (Sokoni; BPF)	CL	20.96	16.67	281,654	0|3213|3745|4139|5548	15,192	100.0%	46.7%	MW342785
3/9/2018	3FB33	Kilifi (Sokoni; BPF)	CL	25.88	21.48	248,816	0|2585|3226|3699|4980	15,192	100.0%	47.0%	MW342783
3/9/2018	3FB34	Kilifi (Sokoni; BPF)	CL	23.93	19.53	287,161	0|2814|3418|3892|5546	15,192	100.0%	47.1%	MW342786
3/9/2018	3FB35	Kilifi (Sokoni; BPF)	OP	27.29	21.48	118,132	0|1245|1422|1573|2224	15,192	100.0%	47.1%	MW342781
3/8/2018	3FB15	Kilifi BPF (Kilifi; BPF)	CL	23.37	18.66	432,551	0|4610|5294|5953|7796	15,192	100.0%	46.5%	MW342798
3/8/2018	3FB16	Kilifi BPF (Kilifi; BPF)	CL	27.92	23.39	676,403	0|7323|8205|9075|11885	15,192	100.0%	46.5%	MW342797
3/15/2018	1MB23	Mombasa (Changamwe; LBM)	CL (AF)	32.7	28.18	933,391	1|9109|10694|12216|16029	15,192	100.0%	46.6%	MW342794
3/16/2018	1MB35	Mombasa (Changamwe; LBM)	CL (AF)	29.12	24.31	455,182	0|4577|5491|6407|9405	15,185 ^e^	100.0%	46.6%	MW342792
3/16/2018	1MB40	Mombasa (Changamwe; LBM)	CL (AF)	29.86	25.18	380,573	0|4080|4664|5252|6662	15,192	100.0%	46.7%	MW342793
3/16/2018	1MB42	Mombasa (Changamwe; LBM)	CL (AF)	29.83	24.66	230,927	0|2241|2671|3023|4125	15,192	100.0%	47.2%	MW342795
1/27/2017	A104	Nairobi (Burma; LBM)	CL (AF)	32.96	33.2	516,967	0|5247|6245|7073|10094	15,192	100.0%	46.6%	MW342805
1/27/2017	A142	Nairobi (Burma; LBM)	OP (AF)	25.27	26.58	237,567	0|735|1110|2578|14486	15,192 ^f^	99.5%	46.7%	MW342806
1/27/2017	A144	Nairobi (Burma; LBM)	OP (AF)	33.67	31.2	395,414	0|1748|2460|4234|26640	15,192	100.0%	46.6%	MW342791
1/27/2017	A148	Nairobi (Burma; LBM)	OP (AF)	21.69	28.51	338,592	0|1368|2271|4082|23996	15,192	100.0%	46.6%	MW342789
1/27/2017	A101	Nairobi (Burma; LBM)	OP (AF)	27.43	30.39	348,356	0|706|1412|3459|26726	15,192	100.0%	46.6%	MW342803
1/27/2017	A105	Nairobi (Burma; LBM)	OP (AF)	28.95	33.45	307,666	1|1433|2306|3486|16181	15,192	100.0%	46.1%	MW342804
1/27/2017	A5136	Nairobi (Kangemi; LBM)	CL (AF)	32.56	35.95	497,720	0|1802|3003|6107|24022	15,192	100.0%	46.2%	MW342807
1/31/2017	A369	Nairobi (Kariobangi North; LBM)	OP (AF)	31.94	34.92	2,275	0|8|13|22|176	15,192	100.0%	46.5%	MW342796
1/31/2017	A371	Nairobi (Kariobangi North; LBM)	CL	32.91	28.14	369,484	0|3604|4275|4889|6842	15,192	100.0%	46.5%	MW342777
1/31/2017	A373	Nairobi (Kariobangi North; LBM)	CL	26.89	22.21	635,518	0|6139|7192|8176|11247	15,192	100.0%	46.5%	MW342779
1/31/2017	A374	Nairobi (Kariobangi North; LBM)	CL	25.47	20.62	616,616	0|5693|6984|8185|11887	15,192	100.0%	46.3%	MW342778
1/31/2017	A375	Nairobi (Kariobangi North; LBM)	CL	24.78	20.86	378,913	0|3629|4389|5030|7902	15,192	99.9%	47.5%	MW342800
1/31/2017	A376	Nairobi (Kariobangi North; LBM)	OP	27.68	22.6	311,605	0|2571|3408|4085|6106	15,192	100.0%	47.5%	MW342776

^a^ This refers to the NGS reads that mapped closest to the NDV reference based on BLASTn of intermediate contiguous sequence for each isolated. ^b^ NDV genomes obtained from allantoic fluids are marked by “(AF)”. ^c^ the numbers for the final consensus sequence length coverage represent distribution (minimum | lower quartile | median | upper quartile | maximum). ^d^ Isolate 34MB09 missing seven bases at the C-terminus, ^e^ Isolate 1MB35 missing 7 bases at the N-terminal, ^f^ Isolate A142/2017 missing bases 1498–1537 and 1602–1643. Abbreviations: OP, oropharyngeal swab; CL, cloacal swab; LBM, live bird market; BPF, backyard poultry farm.

## Data Availability

The complete or nearly complete genome sequences of the 32 Kenyan NDV isolates identified in the current study have been deposited in GenBank with accession number MW342776 - MW342807.

## Supplementary Materials

The following are available online at https://www.mdpi.com/1999-4915/13/1/103/s1, Figure S1: Nucleotide identities between the fusion gene sequences of the NDV isolates sequenced in the current study and related isolates in GenBank. Analyses were conducted using the Maximum Composite Likelihood model [47]. The analysis involved 53 nucleotide sequences from genotypes V (subgenotypes V.1, V.2, and V.3) and VI. Codon positions included were 1st+2nd+3rd+Noncoding. All positions with less than 95% site coverage were eliminated. That is, fewer than 5% alignment gaps, missing data, and ambiguous bases were allowed at any position. Evolutionary analyses were conducted in MEGA X [37], Figure S2: Phylogenetic analyses of the HN gene sequences of the Kenyan NDV isolates reported in the current study in comparison to other isolates in GenBank: (A) full-length nucleotide sequence and (B) amino acid substitutions at different positions within the antigenic sites/neutralizing epitopes of the translated amino acid sequences. The sequences of the 20 and 12 NDV isolates isolated from the LBMs and BPFs are shown in blue and red bold fonts, respectively. Panel B shows only the sites in the Kenyan isolates that contained substitutions. No substitutions were observed in the sialic acid-binding site. Dots indicate sequence identical to the consensus sequence. The evolutionary history was inferred by using the ML method based on the GTR model [47]. This analysis involved 51 nucleotide sequences. Codon positions included were 1st+2nd+3rd+Noncoding. All positions with less than 95% site coverage were eliminated, i.e., fewer than 5% alignment gaps, missing data, and ambiguous bases were allowed at any position (partial deletion option). Evolutionary analyses were conducted in MEGA X [37], Figure S3: Phylogenetic analysis based on the full-length nucleotide sequence of the fusion gene of selected isolates representing all NDV sub/genotypes (*n*=150), which were retrieved from the NDV consortium repository [19]. The 32 Kenyan subgenotype V.3 NDVs reported in the current study are highlighted based on their origins, i.e. LBM (20 sequences in blue color) and BPF (12 sequences in red color). The taxa names for each representative sequence the GenBank accession number, name of the host, country of isolation, strain designation, year of isolation, and the sub/genotype (in Roman numerals). The evolutionary history was inferred by using the Maximum Likelihood method in MEGA X [37] based on the GTR model with 1000 bootstrap replicated. The tree with the highest log likelihood (-26606.2954) is shown. A discrete Gamma distribution was used to model evolutionary rate differences among sites and the rate variation model allowed for some sites to be evolutionarily invariable. The phylogenetic tree is drawn to scale, with branch lengths measured in the number of substitutions per site, Figure S4: Dispersal of NDVs across Kenyan counties between 2010 and 2018. The viruses appear to be dispersed along the various live bird trade routes and markets. Refer to Figure 4 and the manuscript text for a description of the color-coded dispersal of the viruses. The lines connecting the sites of the virus source represent modeled dispersal routes of the viruses, Table S1: Description of the 21 LBMs where indigenous chickens were sampled from in the current study. Birds sold at the indoor LBMs are housed in wire-mesh cages (8–20 birds per cage) throughout Table S2, Estimates of evolutionary divergence between the full-length nucleotide sequence of the fusion gene of the new Kenyan subgenotype V.3 and representative sequences of other isolates (*n*=58). Each representative sequence is shown with the GenBank accession number, name of the host, country of isolation, strain designation, year of isolation, and the sub/genotype (in Roman numerals). The number of base substitutions per site from between sequences are shown. Analyses were conducted using the Maximum Composite Likelihood model. Codon positions included were 1st+2nd+3rd+Noncoding. All positions with less than 95% site coverage were eliminated. That is, fewer than 5% alignment gaps, missing data, and ambiguous bases were allowed at any position. There were a total of 1662 positions in the final dataset. Evolutionary analyses were conducted in MEGA X [37].
